# The feasibility of using Apple's ResearchKit for recruitment and data collection: Considerations for mental health research

**DOI:** 10.3389/fdgth.2022.978749

**Published:** 2022-11-01

**Authors:** Leah Bührmann, Tom Van Daele, Alina Rinn, Nele A. J. De Witte, Dirk Lehr, Jiska Joëlle Aardoom, Lisa Loheide-Niesmann, Jan Smit, Heleen Riper

**Affiliations:** ^1^ Department of Clinical, Neuro & Developmental Psychology, Faculty of Behavioural and Movement Sciences, VU Amsterdam, Amsterdam, Netherlands; ^2^ Amsterdam UMC, Location VUMC, Department Psychiatry, Amsterdam Public Health Research Institute, Amsterdam, Netherlands; ^3^ Expertise Unit Psychology, Technology & Society, Thomas More University of Applied Sciences, Antwerp, Belgium; ^4^ Department of Health Psychology and Applied Biological Psychology, Leuphana University, Lüneburg, Germany; ^5^ Department of Public Health and Primary Care, Leiden University Medical Center, Leiden, Netherlands; ^6^ National eHealth Living Lab, Leiden, Netherlands; ^7^ Behavioural Science Institute, Radboud University, Nijmegen, Netherlands; ^8^ Turku University of Medicine, Turku, Finland

**Keywords:** mHealth, mental health, behavioural activation, Researchkit, app store trial, feasibility, recruitment, dissemination

## Abstract

In 2015, Apple launched an open-source software framework called ResearchKit. ResearchKit provides an infrastructure for conducting remote, smartphone-based research trials through the means of Apple's App Store. Such trials may have several advantages over conventional trial methods including the removal of geographic barriers, frequent assessments of participants in real-life settings, and increased inclusion of seldom-heard communities. The aim of the current study was to explore the feasibility of participant recruitment and the potential for data collection in the non-clinical population in a smartphone-based trial using ResearchKit. As a case example, an app called eMovit, a behavioural activation (BA) app with the aim of helping users to build healthy habits was used. The study was conducted over a 9-month period. Any iPhone user with access to the App Stores of The Netherlands, Belgium, and Germany could download the app and participate in the study. During the study period, the eMovit app was disseminated amongst potential users *via* social media posts (Twitter, Facebook, LinkedIn), paid social media advertisements (Facebook), digital newsletters and newspaper articles, blogposts and other websites. In total, 1,788 individuals visited the eMovit landing page. A total of 144 visitors subsequently entered Apple's App Store through that landing page. The eMovit product page was viewed 10,327 times on the App Store. With 79 installs, eMovit showed a conversion rate of 0.76% from product view to install of the app. Of those 79 installs, 53 users indicated that they were interested to participate in the research study and 36 subsequently consented and completed the demographics and the participants quiz. Fifteen participants completed the first PHQ-8 assessment and one participant completed the second PHQ-8 assessment. We conclude that from a technological point of view, the means provided by ResearchKit are well suited to be integrated into the app process and thus facilitate conducting smartphone-based studies. However, this study shows that although participant recruitment is technically straightforward, only low recruitment rates were achieved with the dissemination strategies applied. We argue that smartphone-based trials (using ResearchKit) require a well-designed app dissemination process to attain a sufficient sample size. Guidelines for smartphone-based trial designs and recommendations on how to work with challenges of mHealth research will ensure the quality of these trials, facilitate researchers to do more testing of mental health apps and with that enlarge the evidence-base for mHealth.

## Introduction

In 2015 Apple launched an open-source software framework called ResearchKit, designed to facilitate medical and health research (1). ResearchKit aims to simplify app development for research purposes by providing a variety of customizable modules to, for example, create informed consent forms, participant reported outcome surveys, and real-time dynamic active tasks (e.g., gait, tapping, spatial memory). This allows for conducting remote, smartphone-based research trials solely through the means of Apple's App Store and tracking and studying the behaviour and wellbeing of individuals who engage with medical and health apps. Such *App Store Trials* (ASTs) can for instance be used for app-based feasibility or effectiveness studies. In ASTs, the intervention under investigation (the app) is hosted by and offered through an open app store, recruitment of participants occurs directly *via* this app store, and data (e.g., demographics of participants, outcome measures) are collected *via* the app. Anyone who installs the app on their device, can function as a potential study participant.

ASTs may have several advantages over conventional trial methods. Research through smartphone applications can, for example, remove geographic barriers, and allow for frequent assessments of participants in real-life settings. ASTs may facilitate study recruitment by reaching more and underrepresented or seldom-heard communities compared to conventional research. On the other hand, it is important to mention that ASTs exclude participants that do not own a smartphone or are unable to use a smartphone. ASTs can further support data collection processes, thereby potentially increasing the amount and quality of the data [e.g., through Ecological Momentary Assessment (2)]. ASTs can be conducted within any app store (e.g., Apple's App Store, Google Play), however, not every app store provider is currently offering the infrastructure, such as ResearchKit, to conduct ASTs.

Numerous apps have been developed with the help of ResearchKit. However, studies have primarily focused on physical conditions [e.g., (3–11)] and ResearchKit-based mental health apps have been developed far less frequently. An examples that uses ResearchKit for the development of a mental health app, is the study by Egger and colleagues (12), in which an app to collect videos of young children with the aims of detecting autism-related behaviours was designed. Egger et al. (12) investigated the acceptability and feasibility of conducting an AST with young children and their caregivers. The entire study procedure was designed with ResearchKit (i.e., e-Consent process, stimuli presentation, data collection) and, over the course of one year, 1,756 families participated in the study by uploading 4,441 videos and completing 5,618 caregiver-reported surveys. The research team concluded that research *via* iPhone-based means was acceptable for their target population. A similar conclusion was drawn by Boonstra and colleagues (13) who investigated the feasibility of using a smartphone app to measure the relationship between social connectivity and mental health. The majority of the 63 participants indicated that data collection (including two mental health questionnaires and an exit survey *via* the app, as well as passively collected data *via* activated Bluetooth) was acceptable and that they would participate in future studies of the investigated app. Further, Byrom et al. (14) tested ResearchKit for delivering a Paced Visual Addition Test and concluded that ResearchKit provided a straightforward approach to app development, that participant acceptance was good and that ResearchKit is a promising tool to enable cognitive testing on mobile devices.

Thus, ResearchKit has shown promise in terms of facilitating the development of and research on (mental) health apps by promoting app-based trials. The field of mental mHealth displays an urgent need for such research, as most applications are currently unguided self-help applications that are directly, and often freely, available to the general public. Platforms such as ORCHA [https://appfinder.orcha.co.uk/] and One Mind PsyberGuide [https://onemindpsyberguide.org] conduct reviews to ensure quality standards and to *provide* transparency on the *quality* of digital health applications. However, most applications are not evidence-based (15) and only few mental health apps have been subjected to effectiveness (16). This is very troublesome for patients in need of selecting self-help and unguided apps.

The aim of the current study was to explore the feasibility of conducting mental health research in the non-clinical population *via* the means of an AST using ResearchKit. In particular, the objectives of this study were to investigate the feasibility of participant recruitment and data collection in this AST. Such feasibility testing is an important prerequisite for studying the effectiveness of app-based research focussing on mental health in the general population. As a test case example, we used an app called eMovit, a behavioural activation (BA) app with the aim of helping users to build healthy habits. Although BA is most commonly associated with the treatment of depression, BA interventions can be adapted to and useful for non-clinical populations as well (e.g., 17–19). eMovit stimulates the development of new and positive behaviours by letting users schedule activities and reminding them on the corresponding days and times.

## Materials and methods

### Ethics

This study was approved by (1) The Scientific and Ethical Review Board (VCWE) of the Faculty of Behavior & Movement Sciences, VU University Amsterdam, The Netherlands, on March 22, 2019 [VCWE-2019-042], the (2) Social and Societal Ethics Committee, KU Leuven, Belgium, on November 23, 2018 [G- 2018 11 1382], and the (3) Ethical Review Board of Leuphana University, Germany, on September 9, 2019 [EB-Antrag 201908-09 Lehr_eMovit]. A timestamped pre-registration can be found at the Open Science Framework [https://osf.io/h6wr4].

### The eMovit app

VU Amsterdam commissioned the development of an BA ResearchKit app and the IT developer Brightfish BV was selected for development. eMovit was designed through an iterative co-design process involving e-mental health experts, IT developers, target users, and pilot participants. eMovit was developed as an example of how to embed a research process within a mHealth application with the aim to study this integrated research process as well as the effectiveness of the app itself. The app was translated from Dutch in three languages – English, German, and the Flemish-Dutch dialect – and released in the Apple App Stores of The Netherlands, Belgium, and Germany.

**Figure 1:**
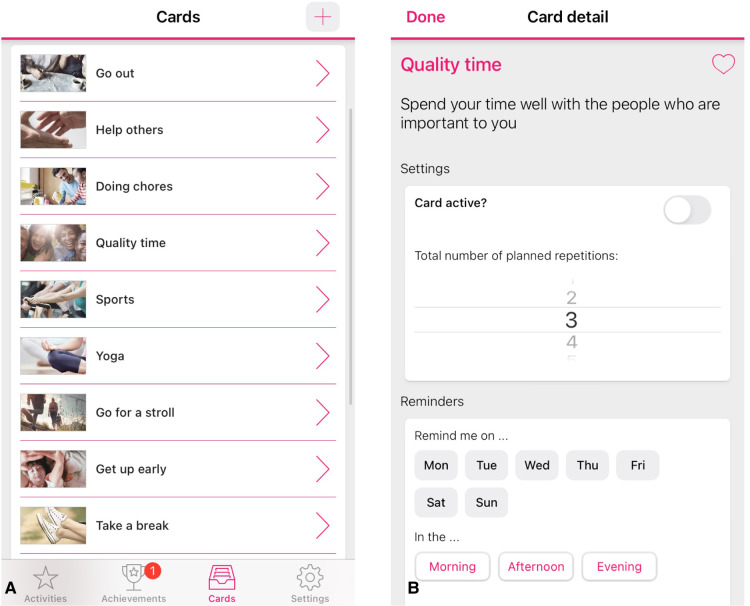
Screenshots of the eMovit app, showing positive activities (**A**), personalisation option of activities (**B**). Note. The eMovit app was developed by Brigthfish BV for VU Amsterdam. © 2022 VU Amsterdam. All rights reserved.

eMovit is a BA intervention app with the aim to activate and build healthy habits of users. The app stimulates the development and maintenance of new and positive behaviours by integrating activity scheduling, reminder setting [e.g., (20, 21)], monitoring (e.g., 22) and rewarding mechanisms (gamification; e.g., 23). More specifically, users can choose from existing positive activities in the app (Figure 1A), create and personalize their own activities, and choose the number of times that they would like to repeat the activity (Figure 1B). Hence, users are free to schedule any number of planned behaviours or activities throughout the day or week, and choose the frequency of which they would like to be reminded of these planned behaviours. Users earn badges and trophies for carrying out these new, positive habits.

### Enrolment, consent and study participation

**Figure 2:**
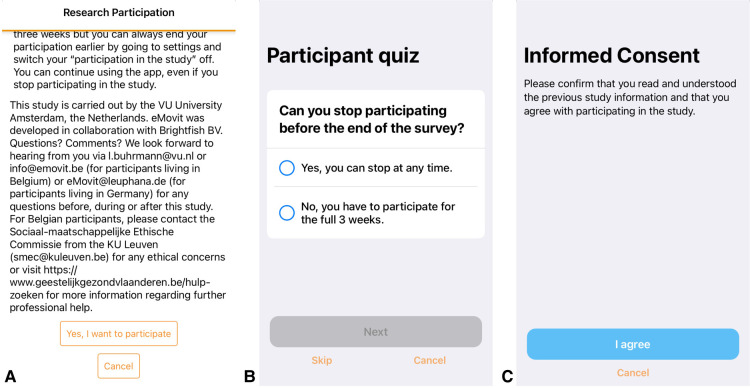
Screenshots of the research information and consent screens of the eMovit app, showing study information (**A**), a 3-item multiple choice participant quiz evaluating their understanding of study participation (**B**), and confirmation of consent (**C**). Note. The eMovit app was developed by Brigthfish BV for VU Amsterdam. © 2022 VU Amsterdam. All rights reserved.

Once participants downloaded the eMovit App, they could self-navigate through welcome information about the functionalities of the App. At the end of this tour, participants could choose whether they wanted to participate in the research study or not. By choosing to participate in research, participants were presented with information about the research, i.e., the study goal, study period, corresponding procedures, anonymity, and researcher contact information (Figure 2A). Users were informed that they would receive two pop-up messages with an invitation to complete the Patient Health Questionnaire-8 (PHQ-8; 24): one at the beginning of the study and one at the end of the study period 3 weeks later. Furthermore, participants were informed that they would receive two questions about their mood and feelings of happiness at three random times each day. After reading the information, participants received a 3-item multiple choice participant quiz evaluating their understanding of study participation, more specifically the research aim, data being shared completely anonymously, and the ability to stop at any time during the study (Figure 2B). In case participants gave the wrong answer, they were provided with the correct answer. After the quiz, participants were asked to confirm whether they had read and understood all the information, and to agree to participate in the research (“I agree” or “Cancel”) (Figure 2C). Study participation was switched on (“I agree”) or off (“Cancel”) accordingly. After participants gave informed consent, they were asked to provide demographics on age, gender, country of residence, employment status, and whether the participant was a twin or triplet. In case participants indicated to be younger than 18 years of age, the study participation was switched off in the app. In case participants indicated to be a twin or triplet, they were asked to complete a number of follow-up questions as part of a different study. After providing the requested information, participants were welcomed to the study and directed to the first questionnaire. The research tools (i.e., participant information, participant quiz, consent form, and questionnaires including notifications/reminders) were programmed using ResearchKit's freely available templates. Information on Covid-19 was included in the App as the onset of the pandemic fell in the study period (see “Procedure”).

### Procedure

The study was conducted over a 9-month period (March 1, 2020–October 31, 2020). As of March 1, 2020, any iPhone user with access to the App Stores of The Netherlands, Belgium, and Germany could download the app and participate in the study. During the study period, the eMovit app was disseminated amongst potential users *via* social media posts (Twitter, Facebook, LinkedIn), paid social media advertisements (Facebook), digital newsletters and newspaper articles, blogposts and other websites. An app landing page was installed to provide potential users with more information [https://emovit.org/]. Participants were recruited online by means of three different strategies: (1) active dissemination using free dissemination strategies, (2) active dissemination using paid dissemination strategies, and (3) passive recruitment (word of mouth and the mere availability of the app in the App Store). Dissemination strategies were designed to either direct potential users to the eMovit landing page [https://emovit.org/] from where a link led to the App Store page [https://Apps.Apple.com/nl/App/emovit], or direct them immediately to the App Store. Using those two pathways was hypothesized to increase download rates as the eMovit landing page provided additional and engaging information. Dissemination strategies were executed by the study partners in Germany (Leuphana University Lüneburg), Belgium (Thomas More University of Applied Sciences), and The Netherlands (VU Amsterdam).

### Outcome measures and data collection

We monitored recruitment using App Store Connect, traffic on the eMovit landing through Matomo Analytics, and interactions with Facebook Ads through Facebook. Participant data and app engagement data was collected by the app itself (ResearchKit, log-files from the developer data base). Matomo Analytics offers full data ownership and protects the user's privacy according to the EU's General Data Protection Regulation (GDPR) and the California Consumer Privacy Act (CCPA) [https://matomo.org/]. App Store Connect is a service by Apple for apps offered in the App Store and complies with Apple's privacy and data protection regulations [https://Appstoreconnect.Apple.com/]. Facebook Ads is a paid service by Facebook that distributes a particular advertisement within the social media platform Facebook and tracks its reach. Facebook adheres to Meta's privacy regulations and GDPR [https://www.facebook.com/privacy/center/]. Table 1 shows the collected outcome data and the corresponding source. ResearchKit provided questionnaire templates which were integrated in the app to collect data on participant demographics and health and wellbeing outcomes. App Store Connect automatically collected data on app analytics such as app units (number of first-time downloads), impressions (total number of app views in the app store), or conversion rate (percentage of impressions that lead to app units).

**Table 1:** Data collected within the eMovit study.

		Source
Recruitment	Product page views	App Store Connect*
	Impressions	App Store Connect*
	App units	App Store Connect*
	Installations	App Store Connect*
	Sessions	App Store Connect*
	Active Devices	App Store Connect*
	Crashes	App Store Connect*
	Deletions	App Store Connect*
	Referrer (App, Web, Campaigns)	App Store Connect*
	Conversion rate	App Store Connect*
Dissemination	eMovit landing page visits	Matomo Analytics
	Facebook activity reach	Facebook Ad
Participants demographics	Age	ResearchKit
	Gender	ResearchKit
	Country of residence	ResearchKit
	Employment status	ResearchKit
	Twin/triplet	ResearchKit
Mood	Current mood	ResearchKit
Happiness	Current feeling of happiness	ResearchKit
Symptoms of depression	PHQ-8	ResearchKit
Log-files	User engagement	Developer data base
User experience/satisfaction	User satisfaction with the App	ResearchKit

* From Apple devices with iOS 8 or higher.

### Data cleaning and analysis

App Store Connect, Matomo Analytics, and Facebook Ads provided descriptive data in a clean and accessible format *via* their user interfaces. This data was extracted from the services and reported as such. The data collected *via* ResearchKit was sourced from the platform, cleaned and prepared for analysis by a statistician (HML). Completion rates on participant demographics, mood, happiness, and symptoms of depression scales were collected. However, the outcome data on these measurements were not analysed and reported on in this publication as the focus of this study was the uptake of and engagement with eMovit. Descriptive statistics of the collected data were analysed using RStudio Version 1.3.1093 (25).

## Results

### Feasibility of participant recruitment in an app store trial

**Figure 3:**
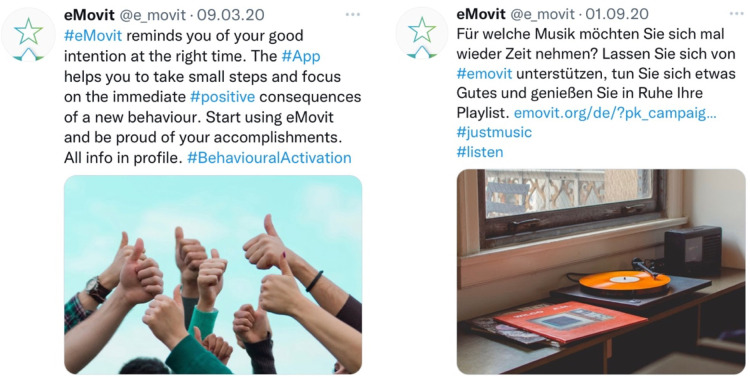
Examples of social media posts (Twitter) to disseminate eMovit.

In terms of active recruitment, eighty-three unpaid dissemination activities were conducted (*n* = 64 by Leuphana University Lüneburg, *n* = 15 by Thomas More, *n* = 4 by VU Amsterdam). These were formulated in lay language and designed to recruit individuals with varying areas of interest to reach the broader population (see Figure 3 for examples of a Twitter tweet in English and German). Furthermore, paid activities were also set up by means of three 2-week Facebook ad campaigns (Table 2). The first part of the first campaign (Phase 1a), directed ad visitors to the eMovit landing page after which they could continue to the App Store. In total, 1,788 individuals visited the eMovit landing page from which 1,170 visitors were generated through specific dissemination campaigns. A total of 144 visitors subsequently entered Apple's App Store through that landing page. The first Facebook campaign (Phase 1a) resulted in no actual app installs, therefore the two-step approach *via* the landing page was changed to lead potential participants directly to the App Store.

**Table 2:** Emovit paid Facebook advertisements: individuals reached (number of individuals the ad was shown to), number of successful conversions (number of times the ad link was clicked) and percentage of conversions compared to the number of individuals reached.

Facebook campaign phase	Country	Individuals	Conversion	Conversion %
1a (July 1–7)	Germany	64,672	565	0.87
	Netherlands	36,560	615	1.68
	Belgium	25,392	310	1.22
	**Total**	**126,624**	**1,490**	**1** **.** **18**
1b (July 8–14)	Germany	71,536	1,002	1.40
	Netherlands	47,554	477	1.00
	Belgium	28,368	175	0.62
	**Total**	**147,458**	**1,654**	**1** **.** **12**
2 (July 29–August 11)	Germany	118,913	3,850	3.24
	Netherlands	77,537	909	1.17
	Belgium	49,904	322	0.65
	**Total**	**246,354**	**5,081**	**2** **.** **06**
3 (September 16–September 29)	Germany	138,717	2,774	2.00
	Netherlands	85,910	1,387	1.61
	Belgium	59,196	932	1.57
	**Total**	**283,823**	**5,093**	**1** **.** **79**
General total	** **	**804,259**	**13,318**	**1** **.** **66**

During campaign phase 1a ad visitors were directed to the eMovit landing page, in campaign phase 1b – 3 ad visitors were directed to the App Store.

### Feasibility of data collection in an app store trial

#### Data collection of dissemination and recruitment activities

**Figure 4:**
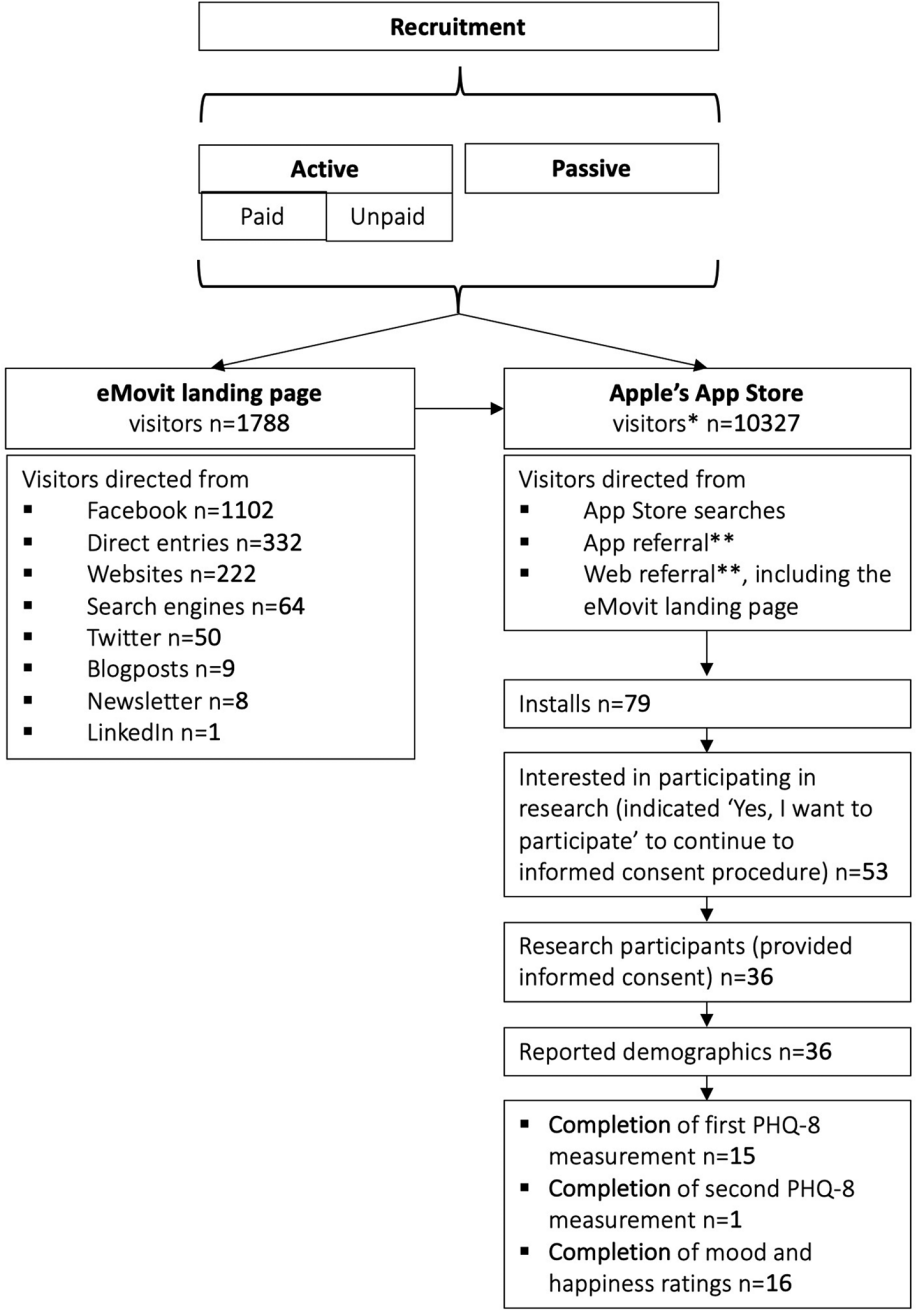
Participant flow. * visitors = app views*;* ** app referral = visitors referred by another app; web referral = visitors referred by a website.

Between March 1, 2020 and October 31, 2020, the eMovit product page was viewed 10,327 times in the App Store, with viewer peaks between July 8–14, July 29–August 11, and September 16–30, 2020. The time periods with the highest numbers of views overlap with the paid Facebook ad campaigns (see Table 2). The participant flow is presented in Figure 4. All in all, eMovit showed a conversion rate of 0.76% (from impressions to app units). Between March 1 and October 31, 2020, App Store Connect reported 12 crashes and 36 deletions of the app, with no more than 2 deletions per day. Nearly half of the eMovit installs were tracked back to App Store searches, followed by app and web referrers, namely Facebook, Instagram, the eMovit landing page, and other websites and campaigns.

#### Data collection from research participants

In total, 53 users indicated that they were interested to participate in the research study by clicking the button “Yes, I want to participate” which led to the informed consent procedure. Of those, 36 consented and completed the demographics and the participants quiz and could be included in the study. Table 3 shows the demographics of the included participants. Fifteen participants completed the first PHQ-8 assessment and only 1 participant completed the second PHQ-8 assessment. A total of 2,180 assessments of current mood and happiness states were offered and 203 of these were completed (response rate of 9.3%; total of *n* = 16). Table 4 shows an overview of the completed measures.

**Table 3:** Demographics of participants.

Demographics		Number of participants (total *n* = 36)
Gender	Male	*n* = 12
	Female	*n* = 19
	Other	*n* = 0
	Information not provided	*n* = 5
Country	The Netherlands	*n* = 10
	Belgium	*n* = 7
	Germany	*n* = 3
	Other	*n* = 12
	Information not provided	*n* = 4
Employment status	Employment fulltime	*n* = 13
	Employment part-time	*n* = 8
	Student/school	*n* = 1
	Homemaker	*n* = 1
	Self-employed	*n* = 4
	Retired	*n* = 4
	Unemployed	*n* = 1
	Unable to work or disabled	*n* = 0
	Information not provided	*n* = 4

**Table 4:** Frequencies of completed research components in the eMovit App.

eMovit study step	Number of participants
Interest in survey	53
Research quiz	36*
E-consent	36
Demographics	36*
PHQ-8 - week 1	15/36
PHQ-8 - week 2	1/36
Mood rating completed	16/36
Happiness rating completed	16/36

* Including questions skipped, but completed.

## Discussion

This study investigated the feasibility of participant recruitment and data collection for conducting mental mHealth research *via* App Store Trials (ASTs) using Apple's ResearchKit, an innovative method to enable large scale mobile-based trials. While it seemed technically straightforward to recruit study participants, reality showed that only 0.76% of product page views resulted in an install and 45.57% of the installs resulted in a completed informed consent form. Drastic decreases in the number of users after initial download is a common phenomenon in ASTs. Chan et al. (26), for example, report a download to informed consent conversion rate of 20.95% with 40,683 initial downloads of their Asthma Health Application. Marketing strategies to promote their app included the development and launch of an app landing page, a partnership with Apple for a launch video and media outreach, press releases and outreach to journalists, co-promotion efforts with asthma advocacy groups, and active social media promotion (Twitter, Facebook). Chan and colleagues (26) explain the high download number by a combination of media publicity and the ease of app download, and the subsequent decrease in numbers from download to informed consent by the “rigor of the consent process” (26, p. 360). Zens and colleagues (4) reached a download to informed consent conversion rate of 57.60% (953 initial downloads) for their ResearchKit app *Back on Track*, which is an outstandingly high conversion rate compared to other ASTs. Reasons for this can be multitude and are not discussed by the authors, however, they do highlight the importance of local language versions of the app to facilitate recruitment and retention rates. Zens and colleagues (4) then report a subsequent low download to active participation conversion of 11.2%, which is comparable to this study with only 18.98% of installs completing the first assessment. Other studies show similar patterns with download to active participation conversion rates around 10% [e.g., (6, 27)]. Chan et al. (26) argue that mobile health developers must understand and incorporate the psychosocial and behavioural needs of mobile users to counteract the steady decrease of user rates from download to informed consent to continued participation (including survey completion) in ASTs. Zens and colleagues (4) support this by suggesting the incorporation of, for example, instant feedback mechanisms, gamification approaches, or the provision of relevant treatment information in mobile health apps.

The low recruitment numbers and conversion rates in the current study could be due to a variety of reasons. Initial download rates may have been influenced by users’ perceived need for a BA app such as eMovit and/or the attractiveness of eMovit to potential users. Because eMovit is a lifestyle app rather than a medical app, the user's gains associated with using the app are more difficult to convey and it is more likely that potential users will not feel the need to engage with such a lifestyle app. Anticipated quality of the app before download could also be a key factor in the users' decision to install the app, this might likely be influenced by the low number of app ratings in the App Store. Additionally, Apple ResearchKit precludes adoption by Android users hereby inherently lowering potential download rates to iOS users only. The low conversion rate from download to informed consent to study participation might have been influenced by the app content (e.g., perceived user-friendliness of the app, the perceived quality of the app), the users’ expectations related to continuously using the app and participating in the study, or users not being willing to provide their data for research (due to privacy reasons or a lack of interest in research). It is beyond the methodology of this study to make a conclusive statement about the reasons for the low recruitment numbers and the sudden drop in participant engagement after the initial download. However, this study offers some considerations and lessons learnt for future mental mHealth research.

### Considerations for future (mental) mHealth research

(1) *The technical incorporation and use of ResearchKit*. ResearchKit provided the research team with the tools to set up a high-quality trial infrastructure. Processes which can be time-consuming or difficult to organize in traditional (online) research, were optimized and easier to implement through ResearchKit. Zens et al. (3) describe ResearchKit as an “easy-to-use framework and powerful tool to create medical studies” (p. 1). This is in line with the current study where the technological incorporation of research components – informed e-consent and data collection tools – by ResearchKit was perceived as fairly straightforward. Templates for creating questionnaires and participant information pages were provided, however, those templates restricted the level of design options for, e.g., number and allocation of text boxes, number of possible words per text boxes. Study information was a prominent component of the study flow and it was possible to easily navigate through the provided information. The corresponding research quiz, a preparation for the e-consent based on the study information, might raise the user's attention towards critical study information in a playful way and enables the researcher to check whether the participant understood the provided study information. The e-consent template, which could easily be incorporated in the flow of the study provides a strong tool to simplify time-consuming paper work for both, the participant and the researchers. We conclude that, from a technological point of view, ResearchKit provides a robust and feasible mean to facilitate the conduct of ASTs.
(2) *App Store Trials using ResearchKit require a well-designed app dissemination process.* Due to the technological advantages ResearchKit offers, the traditional participant recruitment process is replaced by disseminating an app. The app is launched in the App Store and every iPhone user with access to Apple's App Store can download the app and partake in the study. In order to recruit participants, the research team needs to bring the attention of potential app users to the app. The launch of the app in the App Store is thereby solely the minimal dissemination strategy. Isolated examples [e.g., (5, 11, 26)] show that this can be enough to boost download rates, however, the launch itself generally does not make an app sufficiently popular and visible. Recruitment *via* app stores is (in most cases) not a self-runner; what we need is effective dissemination. Dissemination is defined as a targeted approach of distributing information to a specific audience (28). Thereby, dissemination strategies need to be tailored to the purposes of the study and the target population. Those parameters define the scope (specific vs. broad, e.g., population with specific health need vs. general population) and might influence the success of the dissemination campaigns. Dissemination processes benefit from careful planning which can be facilitated by using a dissemination framework as underlying guidance. An overview of existing dissemination frameworks can be found online [https://dissemination-implementation.org/viewAll_di.aspx].
(3) *The issue with sampling from the “social media population”.* While recruiting through social media has many advantages such as low costs and wide reach, it is important to take a critical look at the representativeness of samples recruited through social media channels. Recruitment from social media often follows the so-called “river” sampling, as did the social media sampling in this study. River sampling is a non-probability sampling approach named as such because researchers using the traffic flow of a web page (here Twitter and Facebook) and “catching some users floating by” (29, p. 137). For reasons such as unequal access to the Internet, differences in users’ preferences for social media use, or age differences in social media use across the population, river sampling is likely to lead to coverage bias. Therefore, it is not possible to build a probability model linking the “river” sample to the general population without knowing the demographic distribution of users of a service and the frequency of use of the service (28). In other words, without a well-known and defined sampling frame, no representative sample can be recruited. In addition, the researcher has little control over the reach of recruitment strategies because they depend on the algorithms of the various social media services (30). Without knowing the algorithms of information distribution in the medium, it will not be possible to design recruitment strategies to reach every potential participant, and even if the algorithm is known, it is probably almost impossible to reach every potential participant of the social media service. This again provides unequal opportunity to participate in the study and therefore a biased representation of, what we defined as, general population. Lastly, self-selection of respondents into the sample is a potential risk to the representativeness of the sample. We conclude that not only do conversion rates through social media recruitment appear to be low across studies, but social media sampling also carries a high risk of sampling bias and therefore should be used with caution as it could confound the science.
(4) *The sheer evidence of need does not guarantee engagement of users.* Evidence suggests that Internet-delivered BA is efficacious in the treatment of depression and in increasing general wellbeing (31, 32). Due to its parsimoniousness nature, BA is a suitable candidate to be delivered through a mobile application. eMovit aimed to reach the general population and was therefore designed in a focused manner, reducing functionalities to the very basics, using simplicity as a strategy to remove barriers and reach many potential users. Despite those enabling pre-conditions, the fact that poor mental health rates are high amongst the population (33), and smartphone-based interventions are promising in decreasing the user's threshold in taking advantage of psychotherapeutic (preventative) treatments, engagement of users in eMovit was low. eMovit is not an isolated case: While it is estimated that around 20.000 mental health apps exist (34), a recent analysis found that most user engagement with mental health apps is focused on only two apps (i.e., Calm and Headspace; 35). Problems with the use of eMovit and other mental health apps could be due to several interacting issues, including the appeal of the app, awareness of the app's existence, and potential users' psychosocial and behavioral barriers to actively engaging with the app (e.g., habits). At its best, a well-designed, user-friendly, and useful app leads to positive feedback, and satisfied users attract and motivate other users. Understanding what factors drive users to engage with apps is critical to harnessing the potential of mental health apps for individuals effectively. This understanding could be achieved by involving the end user in the app development process, thereby addressing implementation issues from the outset.
(5) *We need guidelines for conducting and reporting App Store (effectiveness) Trials.* Planning and conducting mental mHealth trials remains novel, despite the clear need for effectiveness testing in mental mHealth and ASTs provide the infrastructure for large-scale research trials. Additionally, the existing literature also shows a need for standardized and comprehensive reporting of ASTs, including detailed descriptions of dissemination/recruitment strategies, to increase transparency, reproducibility and understanding of those trials. Guidance for the evaluation of eHealth exists (e.g., eHealth methodology guide; 36), however, mHealth research encounters unique challenges. Guidelines on AST trial design and reporting as well as recommendations on how to work with challenges of mHealth research might facilitate researchers to do more testing of mental health apps, ensure the quality of these trials, and with that enlarge the evidence-base for mHealth. In turn, the end user would benefit from more effective treatment options and assistance in selecting an appropriate and evidence- based application.

### Study limitations

This AST was based on ResearchKit, which precludes participation from potential participants without an iPhone. Currently, iOS has a market share of 39.17% in Germany, 43.66% in Belgium, and 42.21% in the Netherlands (37). This, coupled with the fact that it is nearly impossible to know the sample frame in social media recruitment, calls into question the representativeness of the sample in this study. The eMovit app, and consequently the implemented dissemination strategies, targeted a general population. Therefore, findings might not generalize to clinical samples since recruitment strategies and conversion rates differ. In addition, we cannot be sure how the COVID-19 pandemic influenced the results since it did cause a rise in mental health problems (38) but also an increased exposure to digital tools (e.g., teleworking), and limitations in which BA activities could be planned (e.g., restrictions in social contact, closing of certain entertainment venues, etc.). It was also beyond the scope of this study to explore why our recruitment results were low, and thus it remains a discussion which adjustments would be necessary to increase participant engagement.

## Conclusion

This study highlights important lessons learnt for future ASTs and mental mHealth research and practice. Apple's ResearchKit provides the means for setting up an infrastructure to conduct ASTs within the field of mental health. With the integration of ResearchKit, eMovit was a valuable tool to facilitate several research processes such as informed e-consent procedures and data collection. It could be easily adapted to different defined populations and therefore used as an add-on in trials to support research processes that are time consuming and costly in traditionally conducted trials. While ResearchKit and the eMovit app hold promise for app-based (effectiveness) research, conversation rates remain low and mHealth research would benefit from structured guidance for setting up ASTs, including well-planned considerations for app dissemination and engagement. Recruitment of participants from online and social media platforms needs to be treated carefully as it might pose a bias to the sampling results and results in weak science.

## Data Availability

The raw data supporting the conclusions of this article will be made available by the authors, without undue reservation.
